# Biomarkers in Pediatric Community-Acquired Pneumonia

**DOI:** 10.3390/ijms18020447

**Published:** 2017-02-19

**Authors:** Nicola Principi, Susanna Esposito

**Affiliations:** 1 Pediatric Highly Intensive Care Unit, Department of Pathophysiology and Transplantation, Università degli Studi di Milano, Fondazione IRCCS Ca’ Granda Ospedale Maggiore Policlinico, Milan 20122, Italy; nicola.principi@unimi.it; 2 Pediatric Clinic, Department of Surgical and Biological Sciences, Università degli Studi di Perugia, Pediatric Highly Intensive Care Unit, Fondazione IRCCS Ca’ Granda Ospedale Maggiore Policlinico, Perugia 06129, Italy

**Keywords:** biomarkers, C-reactive protein, interferon-γ protein-10, procalcitonin, tumor necrosis factor-related apoptosis-inducing ligand

## Abstract

Community-acquired pneumonia (CAP) is an infectious disease caused by bacteria, viruses, or a combination of these infectious agents. The severity of the clinical manifestations of CAP varies significantly. Consequently, both the differentiation of viral from bacterial CAP cases and the accurate assessment and prediction of disease severity are critical for effectively managing individuals with CAP. To solve questionable cases, several biomarkers indicating the etiology and severity of CAP have been studied. Unfortunately, only a few studies have examined the roles of these biomarkers in pediatric practice. The main aim of this paper is to detail current knowledge regarding the use of biomarkers to diagnose and treat CAP in children, analyzing the most recently published relevant studies. Despite several attempts, the etiologic diagnosis of pediatric CAP and the estimation of the potential outcome remain unsolved problems in most cases. Among traditional biomarkers, procalcitonin (PCT) appears to be the most effective for both selecting bacterial cases and evaluating the severity. However, a precise cut-off separating bacterial from viral and mild from severe cases has not been defined. The three-host protein assay based on C-reactive protein (CRP), tumor necrosis factor-related apoptosis-inducing ligand (TRAIL), plasma interferon-γ protein-10 (IP-10), and micro-array-based whole genome expression arrays might offer more advantages in comparison with former biomarkers. However, further studies are needed before the routine use of those presently in development can be recommended.

## 1. Introduction

In the past thirty years, the incidence of community-acquired pneumonia (CAP) in the pediatric population has significantly decreased worldwide, mainly because of the increasingly widespread use of effective means of prevention such as *Haemophilus influenzae* type b vaccine (Hibv) and pneumococcal conjugate vaccines (PCVs) [1,2]. Nonetheless, CAP remains a common cause of pediatric morbidity and mortality, although mortality varies between the industrialized and developing world. In developing countries, where access to care is limited, and interventions that have improved care in the industrialized world are scarce, more than 150 million CAPs are diagnosed in children annually, and a relevant portion of these patients, particularly the youngest, have severe disease and die. Estimates for 2015 have indicated that approximately 1 million children younger than 5 years of age have died from CAP worldwide, and the majority of these deaths have occurred in the developing world, mainly in South Asia and the sub-Saharan area [3]. In industrialized countries, where it has been calculated that, every year, a total number of approximately 4 million CAP cases are diagnosed, death occurs mainly in children with severe chronic underlying conditions, such as lung disease of prematurity, congenital heart disease, and immunosuppression [4]. However, many of these pediatric CAPs are hospitalized, thus affecting the healthcare system. It has been estimated that, in the USA during 2010–2012, several years after the inclusion of Hibv and PCVs in the recommended pediatric immunization schedule, the annual pediatric hospitalization rate for CAP was 15.7 cases per 10,000 children, and the highest rate occurred among subjects younger than 2 years of age (62.2 cases per 10,000 children) [5].

CAP is an infectious disease caused by bacteria, viruses, or a combination of these infectious agents. Moreover, the severity of the clinical manifestations of CAP significantly varies [6]. Consequently, both the differentiation of viral from bacterial CAP cases and the accurate assessment and prediction of disease severity are critical to the effective management of individuals with CAP, including the decision to prescribe antibiotics and to admit patients to the hospital. In adults, both of these goals can be achieved much more easily than in children, particularly those in the first years of life. The limited possibility of obtaining lower respiratory tract secretions or sputum in young children, given their poor tussive force and inability to expectorate, are the most important barrier to obtaining adequate respiratory specimens for etiologic identification through microbiological methods in younger patients [7]. Moreover, whereas in adults several well-established severity scores using clinical findings have facilitated treatment and disposition decisions [8,9,10], no validated clinical severity score is available in children. Only recently, three risk models that appear to accurately estimate risk for severe CAP in children have been proposed. However, these models do not include non-hospitalized children and require an external validation in an expanded population before they can be used in routine practice [11]. Finally, to solve questionable cases, several biomarkers capable of suggesting the etiology and severity of CAP have been studied and used in adult clinical practice and have provided further improvements toward solving CAP diagnostic and therapeutic problems [12,13]. Unfortunately, only a few studies have examined the roles of these biomarkers in pediatric practice, and in most cases, attempts to assess their role in CAP management have produced contrasting results, mainly because of the uncertainty regarding the true etiology of the studied diseases. The main aim of this paper was to describe the present knowledge regarding the use of biomarkers for diagnosing and treating CAP in children, analyzing the most recently published studies of relevance.

## 2. Differentiation of Bacterial from Viral CAP

Initially, most of studies planned to evaluate the roles of biomarkers in defining the etiology of pediatric CAP have been carried out using, alone or in combination, the erythrocyte sedimentation rate (ESR), white blood cell (WBC) count, neutrophil percentage (NP), and serum C-reactive protein (CRP) concentration [14,15,16,17,18,19,20,21,22,23,24]. Some years later, serum procalcitonin (PCT) was also included [25,26,27]. Most of the older studies have been hampered by an incomplete etiologic approach, because only a limited number of infectious agents were assessed, or insensitive techniques were used. Consequently, those studies have not reported reliable information on the use of biomarkers for differentiating bacterial from viral CAP in children. However, over time, the improvement of methods for identifying pathogens has significantly increased pediatricians’ ability to establish the CAP etiology, and more reliable results have been obtained. In more recent studies, a large portion of the data concerning the WBC count, NP, and ESR have indicated that these biomarkers have a poor ability to identify bacterial and nonbacterial pediatric CAP cases, because the proportion of patients with increased values does not substantially differ between bacterial and nonbacterial CAP [20,21]. Regarding CRP, the available data are conflicting and frequently difficult to interpret. In some studies, CRP has not been found to offer any diagnostic advantage, whereas in others, despite the mean values of bacterial CAP being significantly higher than those in viral cases, relevant overlapping has been found, thus reducing the impact of biomarker determination. Moreover, from the various studies, very different threshold values for the identification of bacterial CAP have been defined, and in some cases, the thresholds were so high that they could be measured in only a very small number of patients, a proportion undoubtedly lower than the proportion of bacterial cases in the general pediatric population with CAP. These limitations are relevant in clinical practice because the determination of CRP in decision-making regarding antibiotic administration appears to be useful only for very few children, generally those who are hospitalized and are eventually admitted to the intensive care unit for severe disease, and for whom antibiotic therapy would have been prescribed independently from the laboratory findings. Some examples can illustrate these conclusions. Virkki et al. have measured the CRP levels in a group of 215 children with CAP precisely defined from a microbiological viewpoint and have found that a significant association between a serum CRP concentration of >80 mg/L and bacterial etiology, although similar CRP values were found in a non-marginal number of patients with documented viral infections [20]. CRP > 80 mg/L was measured in 52% of children with bacterial disease and in 28% of children with viral disease (*p* = 0.001). The CRP specificity was 0.72%, but the sensitivity was only 0.52%, a value considered by the authors to be too low for use in clinical practice. Moreover, when the patients were subdivided into two age groups (<2 years and ≥2 years), a CRP concentration of >80 mg/L significantly predicted bacterial CAP only in the younger age group (*p* = 0.003). In a meta-analysis performed by Flood et al., in which 8 studies were considered, involving a total of 1230 children with CAP [22], serum CRP concentrations exceeding 35–60 mg/L were considered a reliable marker of bacterial disease because these values can be measured significantly more often in patients with CAP associated with a bacterial infection than in those with a nonbacterial disease (odds ratio [OR] = 2.58, 95% confidence interval [CI] = 1.20–5.55). However, when the mean OR from the complete meta-analysis was used as the positive likelihood ratio, the positive predictive value (PPV) of the serum CRP exceeding 40–60 mg/L was only 64%.

The results obtained from studies that have tested PCT are slightly better. However, in that case, some negative results have been reported. Moreover, frequently, when a certain degree of PCT ability to identify bacterial cases is evidenced, the cut-off level is found to be very high, thus exhibiting the same limitations previously cited for CRP. Korppi et al. have performed two different studies to evaluate the role of PCT in children with CAP. In the first investigation, the authors found no differences between pneumococcal, *Mycoplasma*/*Chlamydia*, viral and unknown etiology infections in terms of PCT values [23]. In the second study [24], they reported that only PCT concentrations ≥1 ng/mL, a value relatively uncommon in children with mild to moderate disease [25], were a reliable marker of bacterial CAP. Positive results with very high cut-off concentrations have also been reported by Toikka et al. [26], who found that a potential association between the bacterial etiology of CAP and PCT values could be demonstrated only in children with PCT concentrations >2 ng/mL. However, it is likely that even lower PCT values may suggest a bacterial etiology and indicate which CAP cases must be treated with antibiotics, as evidenced by results from the studies in which PCT was used to guide antibiotic treatment. In adults, in the majority of patients with CAP and a PCT concentration <0.5 ng/mL, antibiotic treatment is not necessary because the disease is probably viral and can spontaneously resolve. Moreover, when drugs are administered because the PCT levels are initially higher, treatment can be withdrawn without substantial risks for the patient when the PCT concentrations become lower than the cut-off value [27]. Data regarding children, despite being limited, are consistent with those collected in adults. Esposito et al. have studied a group of 310 children with uncomplicated CAP who were randomized 1:1 to be treated according to PCT values or standard guidelines [25]. The children in the PCT group did not receive antibiotics if their PCT levels at admission were <0.25 ng/mL, and those receiving antibiotics from the time of admission were treated until their PCT levels were ≥0.25 ng/mL. In comparison with the controls, the PCT group received significantly fewer antibiotic prescriptions (85.8% vs. 100%; *p* < 0.05) were exposed to antibiotics for a shorter time (5.37 vs. 10.96 days; *p* < 0.05), and experienced fewer antibiotic-related adverse events (3.9% vs. 25.2%; *p* < 0.05), regardless of the CAP severity. There were no significant between-group differences in outcomes, including the recurrence of respiratory symptoms and a new antibiotic prescription in the month after enrollment.

Data collected in studies in which PCT was compared to CRP appear to indicate that PCT is better than CRP in defining CAP etiology in children, although the cut-off level chosen for comparison can significantly affect the data evaluation, as evidenced by Toikka et al. [26] and Moulin et al. [28]. Toikka et al. have found that, when using PCT > 2 ng/mL and CRP > 150 mg/L, the specificity was >80% for bacterial CAP for both biomarkers, whereas the sensitivities were 50% for PCT and 31% for CRP [26]. Moulin et al. have reported that PCT with a threshold of 2 ng/mL was more sensitive and specific and had greater positive and negative predictive values than did CRP with a threshold of 60 mg/L [28]. In that study, the performance of the tests was evaluated by plotting a receiver operating characteristics curve (ROC) expressed in term of sensitivity, specificity and likelihood ratio (LR). The calculated areas under the curves (AUC) were significantly different (*p* = 0.04) in favor of PCT (0.93 for PCT [95% CI 0.85–0.97] and 0.84 for CRP [95% CI 0.73–0.91]). A slight superiority of PCT in comparison to CRP has also been reported by Esposito et al. in a study involving 433 otherwise healthy children with CAP [29]. The authors conclude that both biomarkers are poor predictors of etiology and found that the AUCs for bacterial CAP of PCT and CRP were 0.69 (95% CI 0.63–0.75) and 0.66 (95% CI 0.61–0.71), respectively.

In recent years, several other biomarkers that had previously been used with satisfactory results in adults have been tested in children. The results have been frequently disappointing, at least when these biomarkers were evaluated alone. A good example in this regard can be found in the studies that have tested lipocalin 2 (LIP2), a protein that is stored in the granules of human neutrophils and has been described as a component of the innate immune system and the acute phase response to infection [30]. Esposito et al. have reported that this biomarker is a poor predictor of CAP etiology in children because the AUC for bacterial disease was found to be very low (0.51 [95% CI 0.40–0.63]), and the sensitivity and specificity were limited to 58.1% and 50.0%, respectively [31]. These findings are quite different from those reported in children by Huang et al. [32], who found that LIP2 was effective for identifying bacterial CAP cases. These contradictory results may be explained by differences in the study populations enrolled in the different investigations. Esposito et al. studied otherwise healthy children [31], whereas Huang et al. evaluated subjects who in many cases had malaria and acquired immunodeficiency infection and malnutrition—all conditions that can significantly modify the LIP2 response [32]. However, these findings, irrespective of their importance in detecting the etiology of CAP, reveal how difficult evaluating a potential biomarker can be and how external factors can modify the results and complicate the final conclusions. Unsatisfactory results have also been obtained in testing of syndecan-4 (SYN4), soluble triggering receptor expressed on myeloid cells-1 (sTREM-1), midregional proatrial natriuretic peptide (MR-proANP), and midregional proadrenomedullin (MR-proADM) [29]. For the detection of bacterial CAP in adult patients, SYN4, a protein that mediates the activity of several inflammatory factors and rapidly increases in response to bacterial infection [33], has been reported to have an AUC of only 0.54 (95% CI 0.42–0.65), with a sensitivity of 31.1% and a specificity of 86.1%—values lower than those evidenced for CRP [34]. Very low AUC values for bacterial CAP identification have also been reported in adults for sTREM-1, a transmembrane glycoprotein expressed on neutrophils, macrophages, and monocytes, which amplifies the inflammatory response and is increased in tissue infected by bacteria and fungi [35], as well as MR-proANP and MR-proADM, which are peptides that have biological actions affecting the cardiovascular system [36]. For all these biomarkers, predictive values for both bacterial and viral CAP of children are lower than that evidenced for CRP and PCT [29].

However, in some cases, new biomarkers, sometimes derived from innovative technologies, have yielded attractive results that may give hope regarding overcoming the present limitations. Myxovirus resistance protein A serum determination and RNA biosignature detection are among these new biomarkers. Myxovirus resistance protein A is a substance induced by type I interferons that is secreted during viral infection and is not present during bacterial infections [37]. It has been evaluated by Engelmann et al., who found that this marker has 96.4% sensitivity and 66.7% specificity for differentiating bacterial and viral infections in children 0 to 16 years old [38]. However, the study sample was too small to draw definite conclusions. Further studies are needed before this marker can be routinely used.

Innovative technologies, including micro-array based whole genome expression arrays, proteomics, and metabolomics, can be a basis for biomarker discovery. For example, the detection of RNA biosignatures has been suggested because bacteria induce specific host responses that can be identified using microarray analyses of blood leukocytes. Mahajuan et al. have evaluated the accuracy of RNA biosignatures in febrile infants 60 days of age or younger and have identified 66 classifier genes distinguishing infants with bacteremia from those without bacterial infections, with 94% sensitivity and 95% specificity [39]. However, this method seems too complicated to be used in routine clinical practice.

Moreover, a possible improvement in the differentiation of bacterial from viral infections may be provided by the combined evaluation of different biomarkers. Although, in most cases, a combination of traditional biomarkers, including WBC count, NP, ESR, CRP, and PCT, has been found to be poorly effective—or not at all effective—in increasing the sensitivity and specificity of a single biomarker [40], it has been presumed that combining new biomarkers with one or more of the traditionally used variables may yield better results. A positive example in this regard comes from a study by Erdman et al. [41], who have investigated children with World Health Organization (WHO)-defined clinical CAP [42] and have found that the combined evaluation of CRP and chitinase 3-like-1, a soluble glycoprotein induced by cytokines and injurious stimuli [43] discriminated between end-point CAP and non-end-point CAP with 93% sensitivity (95% CI 76.5–98.8), 80.8% specificity (95% CI 72.6–87.1), a positive likelihood ratio of 4.9 (95% CI 3.4–7.1), a negative likelihood ratio of 0.083 (0.022–0.32), and a misclassification rate of 0.20 (standard error of 0.038).

Interesting as well are the results of a study carried out by van Houten et al. [44], who tested the combination of CRP, tumor necrosis factor-related apoptosis-inducing ligand (TRAIL), and plasma interferon-γ protein-10 (IP-10). These new markers were chosen because TRAIL is a type II transmembrane protein belonging to the TNF superfamily, which is involved in infection control and in the regulation of both innate and adaptive immune responses [45]. Moreover, IP-10 is a chemokine that is expressed by antigen-presenting cells in response to IFN-γ and attracts activated T-cells to foci of inflammation and has been shown to be involved in the response to bacterial infections [46]. The authors found that this assay is able to distinguish bacterial from viral infections with a sensitivity of 86.7% and a specificity of 91.1%. This assay is significantly more effective than PCT in identifying both bacterial and viral cases because it improves the diagnostic classification for viral and bacterial cases of 6.3% and 5.4%, respectively. However, when compared with CRP alone, the combined assay has been found to improve the identification of patients with viral infection (8.6%) but to be similarly effective in classifying bacterial cases.

## 3. Evaluation of the Severity of Pediatric CAP

Some of the biomarkers used for etiologic diagnosis in children with CAP have been tested to evaluate the disease severity in the same population. PCT has been the most frequently studied, and most of the data, as previously reported in adult patients [47], appear to indicate that the higher the PCT concentration, the higher the risk is that CAP might necessitate hospitalization, cause a prolonged hospital stay, or undergo a negative evolution [48,49]. Moreover, PCT has been found to be superior to CRP in the severity assessment. Lee et al. have reported an increase in both PCT and CRP in lobar CAP with respect to bronchopneumonia but have found that PCT has better sensitivity and specificity in detecting CAP with a radiological finding suggesting increased severity [50]. However, in a very recent publication, the superiority of PCT over CRP has been found to no longer be evident [51].

Other biomarkers for which the correlation with CAP severity in children has been assessed are IL-6, MR-proADM, and copeptin (CoPEP). Interleukin-6 (IL-6) is the only one of 15 serum cytokines studied that is correlated with indicators of disease severity in childhood CAP, including lobar consolidation [52]. Regarding MR-proADM, it has been shown that children with severe CAP arising from bacteremia or empyema, have significantly higher values of this biomarker than do children with mild, uncomplicated CAP (0.18 vs. 0.08 nmol/L; *p* = 0.039). By contrast, conflicting results have been reported for CoPEP. A study by Alcoba et al. has concluded that this biomarker cannot distinguish complicated from uncomplicated CAP cases (*p* = 0.95) [53], whereas Abdel-Fattah et al. have reported that significantly higher median serum CoPEP levels (*p* = 0.03) in children with CAP than in healthy controls and in those who died (*p* = 0.04) [54], thus supporting the use of this biomarker for identifying children with a potential poor outcome. Finally, in *Mycoplasma pneumoniae* CAP, lactate dehydrogenase (LDH) has been found to be a good predictor of refractory infection [54]. ROC curve analysis has indicated an AUC of LDH of 0.718 with a cut-off of 379 IU/L, a value higher than that of ESR with a cut-off of 32.5 mm/h.

However, for all these biomarkers, including those with positive results, the low sample size of investigated children and the lack of defined threshold values capable of indicating which patients really need hospitalization for increased risk of negative evolution makes them poorly effective in clinical practice.

## 4. Conclusions

Despite several attempts to differentiate bacterial from viral disease and predict severity and outcome, the etiologic diagnosis of pediatric CAP and the estimation of potential outcomes remain unsolved problems in most cases. The use of biomarkers adds little to what can be derived from the evaluation of clinical and radiological signs and symptoms, at least when the various tests are used alone. Among traditional biomarkers, PCT appears to be the most effective both in selection of bacterial cases and in evaluation of severity. However, a precise cut-off level able to separate bacterial from viral cases and mild from severe cases has not been defined. Further studies in this regard are required, given that the determination of this marker is easy and rapid tests for use in the community have been developed. The three-host protein assay based on CRP, TRAIL, and IP-10 and micro-array-based whole genome expression arrays might offer more advantages than do former biomarkers. However, further studies are necessary before routine use of biomarker assays presently in development can be recommended.
